# Unusual neurological manifestations of bilateral medial medullary infarction: A case report

**DOI:** 10.1515/med-2021-0382

**Published:** 2022-01-03

**Authors:** Ruizhi Zheng, Ting Zhang, Xianzhu Zeng, Miao Yu, Zhao Jin, Jing Zhang

**Affiliations:** Department of Neurology, The Third Central Clinical College of Tianjin Medical University, Tianjin 300170, China; Tianjin Key Laboratory of Extracorporeal Life Support for Critical Diseases, Tianjin, China; Artificial Cell Engineering Technology Research Center, Tianjin, China; Tianjin Institute of Hepatobiliary Disease, Tianjin, China; Department of Neurology, The Third Central Clinical College of Tianjin Medical University, Tianjin 300170, China; Tianjin Key Laboratory of Cerebrovascular and Neurodegenerative Diseases, Tianjin Dementia Institute, Department of Neurology, Tianjin Huanhu Hospital, Tianjin 300350, China

**Keywords:** bilateral medial medullary infarction, magnetic resonance imaging, diffusion-weighted image, fetal-type posterior cerebral artery, bilateral facial paralysis

## Abstract

Bilateral medial medullary infarction (BMMI) is an extremely rare type of cerebrovascular accident often resulting in poor functional consequences. “Heart appearance” on diffusion-weighted imaging (DWI) of magnetic resonance imaging (MRI) is the unique presentation of BMMI. In this article, we present an acute ischemic stroke patient whose brain MRI showed the atypical “heart appearance” sign, manifested unusual bilateral central facial paralysis concurrently. For an early diagnosis of BMMI, it is essential to recognize the characteristic clinical and MRI findings of this rare type of stroke. Abnormal small dot or linear DWI signal at the midline of the brainstem should not be ignored at the early stage of stroke.

## 1 Introduction

Medial medullary infarction (MMI) accounts for only less than 1–1.5% of ischemic strokes in the posterior circulation [1,2], in which bilateral infarctions are even rare. BMMI usually presents with acute onset of dysarthria, dysphagia and quadriplegia commonly sparing the face [3]. The progression in MRI technology plays an essential role in the differentiation with brainstem encephalitis and Guillain-Barre’s syndrome [4]. Although the characteristic “heart appearance” or “Y-shaped” sign is well recognized by neurologists, abnormal atypical signs on MRI must be noted as well. The aim of this article was to describe a rare case, where the patient had BMMI with bilateral central facial paralysis.

## 2 Case presentation

A 53 year old Chinese male machine operator presented with acute quadriplegia and numbness in his upper and lower extremities symmetrically on April 24, 2016. He also complained of noticeable slurred speech and difficulties in swallowing liquids. Droopy eyelids, double vision and vertigo were all denied. Past medical history included untreated hypertension for 10 years. He had a moderate alcohol consumption for 30 years; however, he was a heavy smoker (75 pack year). The suspected diagnosis was an acute ischemic stroke. Unfortunately, the initial manifestations appeared 14 h before he was admitted to the hospital so that he had missed the window period for intravenous recombinant tissue plasminogen activator (rt-PA). Thereafter, he was treated with tablet clopidogrel 75 mg, atorvastatin 20 mg daily and intravenous argatroban 10 mg twice a day.

On admission, the blood pressure was 178/90 mmHg, pulse was 68/min, and body temperature was 37.0°C. He was lucid but had dysarthria. Cranial nerve examination revealed normal pupils and eye movement. The pinprick and temperature sensations in the distribution of all three branches of the trigeminal nerve bilaterally were normal. There was central facial palsy on both sides; nevertheless, without loss of taste sensation in the anterior two-thirds of the tongue. The arch of the soft palate was paralyzed on both sides, while the absence of gag reflex and hoarseness were noted. The examinations of other cranial nerves were normal. Muscle strength revealed 0/5 on the Medical Research Council (MRC) scale in the four extremities. On this basis, tests of cerebellar function, coordination and balance were unable to perform. The perception of pinprick, proprioception and vibration sensations were all normal symmetrically. Deep tendon reflexes were 1+ in upper limbs and 2+ in lower limbs bilaterally, with Babinski’s sign, Chaddock’s sign and Oppenheim’s sign being positive on both sides. His National Institutes of Health Stroke Scale (NIHSS) score was 16/42.

**Figure 1:**
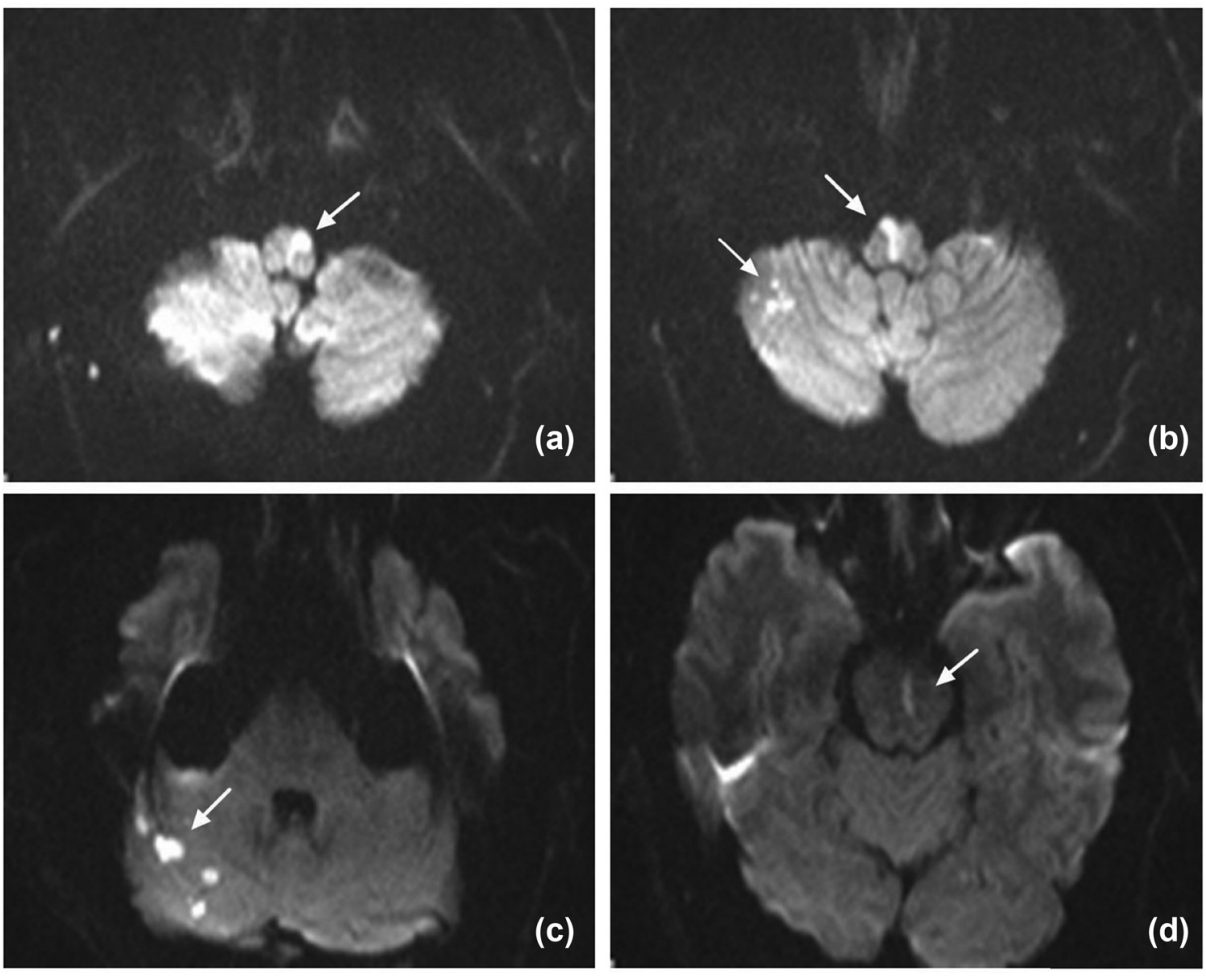
(Axial DWI imaging): (a and b; arrow) bilateral medial medullary infarction involves the ventral, middle parts of the medulla oblongata, which were compatible with atypical “heart appearance.” (b and c) The right cerebellar hemisphere and (d) pons also showed high-intensity lesions.

The initial brain MRI was performed 10 h after the onset of the symptoms. The consecutive horizontal sections were noted on diffusion-weighted MRI at 3.0T, a defined hyperintensive linear signal at right anterior-medial territory with a dot-like signal on the other side, which disclosed an atypical “heart-shaped” hyperintensity in the medial medulla (Figure 1a and b). In addition to the medulla oblongata, the infarcted areas also included pons and the right cerebellar hemisphere. Axial T2-weighted MRI image showed hyperintensive signal, while apparent diffusion coefficient (ADC) image (axial) showed hypointensity in the same region. Meanwhile, the signals remained normal on fluid-attenuated inversion recovery (FLAIR).

**Figure 2:**
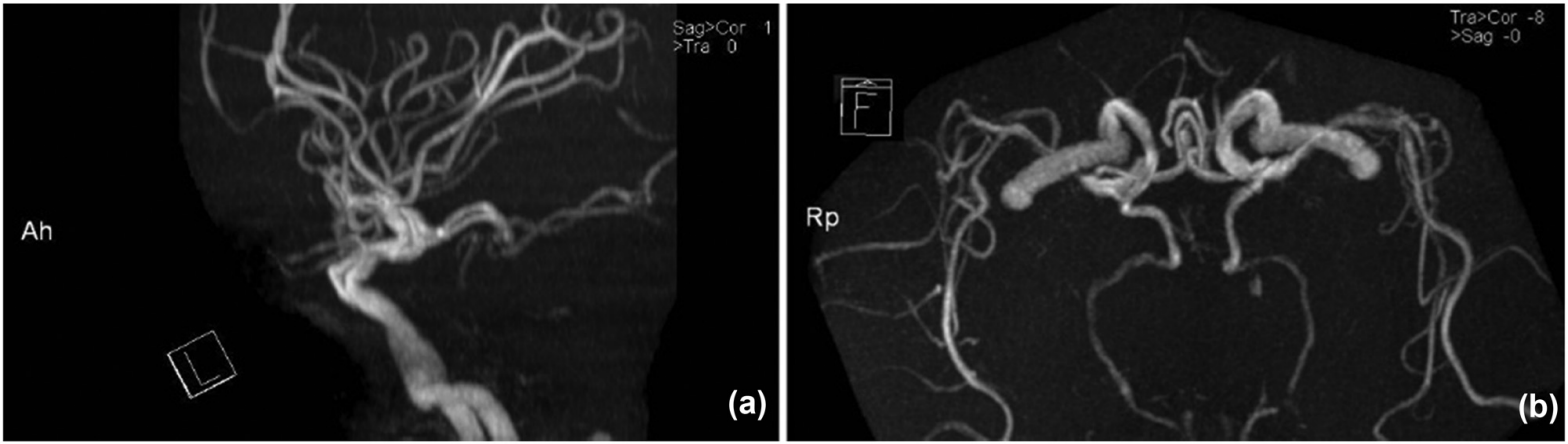
(TOF-MRA imaging): the lateral projection of an MRA image shows the hypoplastic BAS. (a and b) The posterior communicating arteries supply the territory of all branches of the PCAs, which were considered as fetal-type. (b) Caudocranial projection image shows that both PCAs were divided from ICA separately.

Brain magnetic resonance angiography (MRA) showed atherosclerosis of the bilateral internal carotid artery (ICA) throughout the entire length. The outlines of the vertebral-basilar system (VBS) were irregular and vague, with its lumen diameter <2 mm. Both the posterior cerebral arteries (PCA) were originated from a posterior communicating artery (PCoA) separately, which were considered as a fetal-type posterior cerebral artery (FTP) variation (Figure 2a and b). Meanwhile, the anterior spinal artery (ASA) was not visualized. There is no apparent dissection of the vertebral arteries (VA). The option of digital subtraction angiography (DSA) and endovascular approach was offered to his relatives, but his wife refused both.

Complete blood counts showed no abnormality. Biochemistry showed elevated levels of fasting blood glucose (FBG, 9.73 mmol/L) and HbA1c (8.5%). Postprandial blood glucose at 2 h fluctuated between 8.17 and 11.32 mmol/L, so he was diagnosed with type 2 diabetes finally and received insulin therapy. Blood lipid analysis showed elevated levels of serum low-density lipoprotein (LDL, 4.84 mmol/L), triglyceride (TG, 1.76 mmol/L) and total cholesterol (TC, 6.81 mmol/L). The plasma tHcy concentration (14.1 μmol/L) was slightly increased. Qualitative urinalysis showed proteinuria (1+) and glucosuria (3+). In addition to normal blood urea nitrogen (BNU, 7.36 mmol/L) and serum creatinine (SCr, 80 μmol/L), the following investigations were normal or negative: liver function tests, creatine kinase (CK), CK-MB isoenzyme, troponin I, B-type natriuretic peptide (BNP), thyroid function tests, vitamin B12 and folate levels. Hepatitis B-surface antibodies (Anti-HBs) and hepatitis B-core antibody (Anti-HBc) were positive, but serology testing including hepatitis B-surface antigen (HBsAg), hepatitis C, HIV and syphilis were all negative. Transthoracic echocardiogram, 24 h-Holter monitoring and routine electrocardiogram were normal.

Based on these clinic-radiological findings, the patient was diagnosed with bilateral medial medullary infarction (BMMI). Although the early medicinal treatment and rehabilitation were performed, signs of bulbar palsy and tetraplegia still remained. The patient had a modified Rankin Scale (mRS) score of 5/6. He was transferred to another hospital for long-term care after 2 weeks.


**Ethics statement:** This is a description of a clinical case with a brief literature review. There was no formal research ethics approval required or no experimental intervention into routine care. Fully informed consent from the patient’s family was obtained.

## 3 Discussion

### 3.1 BMMI

BMMI is a rare subtype of all ischemic strokes, which usually presents with hypoglossal palsy, quadriplegia, loss of deep sensation and bulbar dysfunction with or without respiratory failure. Pathognomonic MRI findings of BMMI reveal a bilateral infarction at levels of the medial medulla, which is well known as the “heart appearance” [2,5,6,7]. As for our patient, it was the linear and dot-like DWI lesions of both sides of the medulla on two consecutive transverse sections altogether that formed the imagiological presentation, which was not as typical as the published literature.

The blood supply of the anterior-medial territory of the medulla oblongata consists of two major parts: the superior one-third is known as the paramedian branches of VA, and the inferior two-thirds revealed the ASA. On account of the interindividual variation of these vastly complex networks and the spatial resolution limitation of MRA, it is commonly difficult to identify the occluded culprit vessel. There is consensus that BMMI commonly results from atherothrombotic involving the vertebral or ASA. In other words, the predominant presumed mechanisms were categorized into large artery atherosclerotic infarction according to the guidelines [8]. Extended thrombosis in the vertebra-basilar junction and the anatomic variability of paramedian perforating from vertebral or ASA; alternatively, one paramedian artery supplies both pyramids, maybe the immediate mechanism of the simultaneous bilateral infraction. This might explain BMMI caused by unilateral VA lesions reported in the literature [9,10,11]. In the largest series of 86 patients with MRI-identified unilateral MMI, Kim and Han [12] reported that large-artery atherosclerosis (62%) is the commonest cause. Pongmoragot et al. [13] also revealed large-artery atherosclerosis involving the VA (38.5%) and basilar artery (BA, 19.2%) accounted for the majority of the vascular pathology in a series of 38 patients with restricted inclusion criteria of BMMI.

It is undeniable that small penetrating artery disease represents another stroke mechanism of atheromatous branch occlusion as well, which could not be commonly demonstrated by MRA or DSA [14]. Pongmoragot et al. also found that more than one-third (38.5%) of the BMMI patients had no abnormal vascular findings. Furthermore, cardiac embolism, dissection of VA [15], even Takayasu arteritis [16] and Fabry disease [17] could be the mechanism of BMMI. To mention our case, the brain MRA showed large-artery atherosclerosis throughout the entire VBS, combined with barely visualized VA and ASA. Therefore, the fetal-type variant of PCA that was supplied by the anterior circulation from the ICA, rather than from the posterior circulation, played an essential compensatory role. Unfortunately, contrast-enhanced supraaortic MRA or DSA is not available for our patient. The precise etiology underlying the stroke remains to be elucidated since current imaging findings are hard to differentiate between the vascular lesion and hypoplasia/aplasia of VA and BA.

### 3.2 Bilateral facial paralysis of the central type

Central facial paralysis is extremely rare in medullary lesions and it is rarer still to bilateral paralysis in view of anatomic reasons. Literarily, MMI has been classically described as Déjerine syndrome, a triad of contralateral hemiparesis, proprioceptive impairment and ipsilateral hypoglossal palsy. However, on reviewing the literature, we found that central facial paralysis, not seen in the classic description, is found in a certain proportion(18–30%) of patients with MMI [12,18]. Bilateral central facial palsy, which has been reported as well [19,20], accounts for less than 16.2% of patients with BMMI [13].

It is well known that the anatomy of the human facial nucleus and peripheral facial nerves have been established. However, the confirmative course of the corticobulbar fibers that connect the motor cortex with the facial nucleus still remains uncertain. The facial corticobulbar tract fibers that arise from the motor cortex provide strongly unilateral innervation to the contralateral lower facial nucleus and bilateral innervation to the upper facial nucleus. Classic symptom localization has postulated that brainstem lesions rostral to the upper mid-pons result in contralateral facial paresis of central type. However, the absence of contralateral upper motor neuron facial palsy due to unifocal ischemic lesions at the medial medulla has been confirmed in the literature [21,22,23]. Transcranial magnetic stimulation combined with lesion topography analysis by MRI was used by Urban [23] to confirm these loop-shaped cortico-facial projections, which reveals facial paresis can occur ipsilateral to the lesion side at levels of upper medulla either. Based on the above-mentioned findings, it has been hypothesized that some of the facial corticobulbar fibers descend to the level of the upper-middle medulla ventromedially, making a loop before they decussate and then ascend to the dorsolateral medulla to supply the contralateral facial nucleus. On the other hand, lateral medullary infarction relatively often results in facial weakness on the side of the lesion [21,22,23]. It has also been postulated that facial weakness on the side of the lesion results from corticobulbar fibers being interrupted while they ascend contralaterally after decussating. Although this aberrant bundle was already described by Currier et al. [24] in the 1960s, conclusive pathological evidence has not been found yet. Interestingly, bilateral central facial paralysis occurs in our patient and suggests that this aberrant bundle, within both sides of the brainstem, does exist.

## 4 Conclusion

BMMI is an unusual stroke type, which is usually related to large arteries or branch disease of posterior circulation and is commonly associated with severe morbidity and mortality. As a result of anatomic variations of corticobulbar fibers in the brainstem, the central type of facial paralysis led by medullary infarction should not be ignored. For an early diagnosis, it is essential to bear in mind the characteristic findings obtained by diffusion-weighted MRI.

## References

[1] Bassetti C , Bogousslavsky J , Mattle H , Bernasconi A . Medial medullary stroke: report of seven patients and review of the literature. Neurology. 1997;48(4):882–90. 10.1212/wnl.48.4.882 9109872

[2] Shono Y , Koga M , Toyoda K , Matsuoka H , Yokota C , Uehara T , et al. Medial medullary infarction identified by diffusion-weighted magnetic resonance imaging. Cerebrovasc Dis. 2010;30(5):519–24. 10.1159/000319887 20861624

[3] Kleinert G , Fazekas F , Kleinert R , Schmidt R , Payer F , Offenbacher H , et al. Bilateral medial medullary infarction: magnetic resonance imaging and correlative histopathologic findings. Eur Neurol. 1993;33(1):74–6. 10.1159/000116906 8440293

[4] Montalvo M , Ali R , Khan M . Bilateral medial medullary infarction presenting as Guillain Barré Syndrome: a diagnostic challenge. J Neurolog Sci. 2015;352(1–2):135–6. 10.1016/j.jns.2015.03.048 25888528

[5] Maeda M , Shimono T , Tsukahara H , Maier SE , Takeda K . Acute bilateral medial medullary infarction: a unique ‘heart appearance’ sign by diffusion-weighted imaging. Eur Neurol. 2004;51(4):236–7. 10.1159/000078549 15159607

[6] Tokuoka K , Yuasa N , Ishikawa T , Takahashi M , Mandokoro H , Kitagawa Y , et al. A case of bilateral medial medullary infarction presenting with “heart appearance” sign. Tokai J Exp Clin Med. 2007;32(3):99–102. 21318946

[7] Gupta A , Goyal MK , Vishnu VY , Ahuja CK , Khurana D , Lal V . Bilateral medial medullary infarction: the ‘heart’ reveals the diagnosis. Int J Stroke. 2014;9(4):E18. 10.1111/ijs.12273 24798043

[8] Amarenco P , Bogousslavsky J , Caplan LR , Donnan GA , Hennerici MG . New approach to stroke subtyping: the A–S–C–O (phenotypic) classification of stroke. Cerebrovasc Dis. 2009;27(5):502–8. 10.1159/000210433 19342826

[9] Zhang L , Zhang GL , Du JM , Ma ZL . Bilateral medial medullary infarction with nondominant vertebral artery occlusion. J Stroke Cerebrovasc Dis. 2015;24(9):e241–4. 10.1016/j.jstrokecerebrovasdis.2015.04.005 26175270

[10] Paliwal VK , Kalita J , Misra UK . Dysphagia in a patient with bilateral medial medullary infarcts. Dysphagia. 2009;24(3):349–53. 10.1007/s00455-008-9194-8 19115072

[11] Tai ML , Katiman E , Rahmat K , Tan CT . Acute bilateral medial medullary infarct with hypoplastic vertebral artery. Clin Neurol Neurosurg. 2012;114(10):1365–7. 10.1016/j.clineuro.2012.03.031 22512947

[12] Kim JS , Han YS . Medial medullary infarction: clinical, imaging, and outcome study in 86 consecutive patients. Stroke. 2009;40(10):3221–5. 10.1161/STROKEAHA.109.559864 19628797

[13] Pongmoragot J , Parthasarathy S , Selchen D , Saposnik G . Bilateral medial medullary infarction: a systematic review. J Stroke Cerebrovasc Dis. 2013;22(6):775–80. 10.1016/j.jstrokecerebrovasdis.2012.03.010 22541608

[14] Zhou ZH , Wu YF , Wu WF , Liu AQ , Yu QY , Peng ZX , et al. Giant “heart appearance-like sign” on MRI in bilateral ponto-medullary junction infraction: case report. BMC Neurol. 2020;20(1):107. 10.1186/s12883-020-01683-7 PMC7092499 32293317

[15] Hagiwara N , Toyoda K , Torisu R , Inoue T , Yasumori K , Ibayashi S , et al. Progressive stroke involving bilateral medial medulla expanding to spinal cord due to vertebral artery dissection. Cerebrovasc Dis. 2007;24(6):540–2. 10.1159/000111220 18042979

[16] Deshpande A , Chandran V , Pai A , Rao S , Shetty R . Bilateral medial medullary syndrome secondary to Takayasu arteritis. BMJ Case Rep. 2013;2013:bcr0120125600. 10.1136/bcr-01-2012-5600 PMC3762468 23943806

[17] Jiang S , Wang L , Yan Y , Zhu Q , Wan J , Sun J , et al. Fabry disease presenting as bilateral medial medullary infarction with a “heart appearance” sign: a case report. BMC Neurol. 2020;20(1):180. 10.1186/s12883-020-01766-5 PMC7216630 32397962

[18] Kameda W , Kawanami T , Kurita K , Daimon M , Kayama T , Hosoya T , et al. Lateral and medial medullary infarction: a comparative analysis of 214 patients. Stroke. 2004;35(3):694–9. 10.1161/01.STR.0000117570.41153.35 14963274

[19] Toyoda K , Imamura T , Saku Y , Oita J , Ibayashi S , Minematsu K , et al. Medial medullary infarction: analyses of eleven patients. Neurology. 1996;47(5):1141–7. 10.1212/wnl.47.5.1141 8909419

[20] Wada K , Gotoh T , Hashimoto Y , Kimura K , Uchino M . A case of bilateral infarction of medial pontomedullary junction. Rinsho Shinkeigaku = Clin Neurol. 1996;36(10):1186–9. 8997147

[21] Cavazos JE , Bulsara K , Caress J , Osumi A , Glass JP . Pure motor hemiplegia including the face induced by an infarct of the medullary pyramid. Clin Neurol Neurosurg. 1996;98(1):21–3. 10.1016/0303-8467(95)00082-8 8681473

[22] Terao S , Takatsu S , Izumi M , Takagi J , Mitsuma T , Takahashi A , et al. Central facial weakness due to medial medullary infarction: the course of facial corticobulbar fibres. J Neurol, Neurosurg, Psychiatry. 1997;63(3):391–3. 10.1136/jnnp.63.3.391 PMC2169694 9328262

[23] Urban PP , Wicht S , Vucorevic G , Fitzek S , Marx J , Thömke F , et al. The course of corticofacial projections in the human brainstem. Brain. 2001;124(Pt 9):1866–76. 10.1093/brain/124.9.1866 11522588

[24] Currier RD , Giles CL , Dejong RN . Some comments on Wallenberg’s lateral medullary syndrome. Neurology. 1961;11:778–91. 10.1212/wnl.11.9.778 13718920

